# Patient engagement as a risk factor in personalized health care: a systematic review of the literature on chronic disease

**DOI:** 10.1186/gm533

**Published:** 2014-02-26

**Authors:** Leigh Ann Simmons, Ruth Q Wolever, Elizabeth M Bechard, Ralph Snyderman

**Affiliations:** 1 Current address: Duke University School of Nursing, DUMC Box 3322, Durham, NC 27710-3322, USA; 2 Center for Research on Prospective Health Care, Duke University Health System, Durham, NC 27710, USA; 3 Current address: Duke Integrative Medicine, DUMC Box 102904, Durham, NC 27710-2904, USA; 4 Department of Psychiatry and Behavioral Sciences, Duke School of Medicine, Durham, NC 27710-2904, USA; 5 Current address: Department of Medicine, DUMC Box 3059, Durham, NC 27710-3059, USA

## Abstract

**Background:**

The role of patient engagement as an important risk factor for healthcare outcomes has not been well established. The objective of this article was to systematically review the relationship between patient engagement and health outcomes in chronic disease to determine whether patient engagement should be quantified as an important risk factor in health risk appraisals to enhance the practice of personalized medicine.

**Methods:**

A systematic review of prospective clinical trials conducted between January 1993 and December 2012 was performed. Articles were identified through a medical librarian-conducted multi-term search of Medline, Embase, and Cochrane databases. Additional studies were obtained from the references of meta-analyses and systematic reviews on hypertension, diabetes, and chronic care. Search terms included variations of the following: self-care, self-management, self-monitoring, (shared) decision-making, patient education, patient motivation, patient engagement, chronic disease, chronically ill, and randomized controlled trial. Studies were included only if they: (1) compared patient engagement interventions to an appropriate control among adults with chronic disease aged 18 years and older; (2) had minimum 3 months between pre- and post-intervention measurements; and (3) defined patient engagement as: (a) understanding the importance of taking an active role in one’s health and health care; (b) having the knowledge, skills, and confidence to manage health; and (c) using knowledge, skills and confidence to perform health-promoting behaviors. Three authors and two research assistants independently extracted data using predefined fields including quality metrics.

**Results:**

We reviewed 543 abstracts to identify 10 trials that met full inclusion criteria, four of which had ‘high’ methodological quality (Jadad score ≥ 3). Diverse measurement of patient engagement prevented robust statistical analyses, so data were qualitatively described. Nine studies documented improvements in patient engagement. Five studies reported reduction in clinical markers of disease (for example HbA1C). All studies reported improvements in self-reported health status.

**Conclusions:**

This review suggests patient engagement should be quantified as part of a comprehensive health risk appraisal given its apparent value in helping individuals to effectively self-manage chronic disease. Patient engagement measures should include assessment of the knowledge, confidence and skills to prevent and manage chronic disease, plus the behaviors to do so.

## Background

Improved models for the delivery of health care in the United States are greatly needed given that more than three-fourths of healthcare expenditures account for treatment of preventable chronic disease [1-4]. Personalized medicine, which emerged as an outgrowth of anticipated predictive capabilities resulting from the sequencing of the human genome, has been highlighted as one solution to the healthcare crisis in the US [5,6]. Initially, personalized medicine was conceptualized as integrating new predictive technologies and tailored therapies into the practice of medicine, so that medicine shifted away from a reactive, disease-oriented approach to one that is predictive, preventive, and personalized [7-10]. As the concept has evolved, partly in response to as yet unfulfilled expectations regarding the availability of genomic-based tools and therapies to prevent and manage chronic disease, increasing attention has focused on how to incorporate all personalized tools - genomic and non-genomic - into clinical care [11]. Indeed, the broader term, 'personalized health care', has been suggested to describe a coordinated, strategic approach to care that applies the concepts of systems biology and personalized, predictive, preventive, and participatory care [11-13]. Central to personalized health care is the development of a personalized health care plan, which is a customized plan of care the provider and patient develop collaboratively based on a comprehensive health status and risk assessment, shared goals, and tracking measures [6,11,12]. The plan serves to simultaneously organize and coordinate care while engaging the patient in the process of care delivery and self-management of health.

The importance of engaging patients in their care is earning increasing attention from clinicians, researchers, and policymakers alike [14-16], because the actions people do - and do not - take are critical for successful prevention and management of disease. An informed, activated patient is essential in the chronic care model, which provides a framework for delivery models focused on chronic disease treatment [17] and prevention of health risk behaviors [18]. The engagement behavior framework [19] suggests that not only do patients benefit from being engaged, but also current healthcare delivery practices implicitly and explicitly demand that patients possess the skills to participate constructively in their care. Clinical models such as the patient-centered medical home [20,21] and prospective health care [5,9,10] highlight the importance of patient engagement in achieving coordinated care, increasing rates of treatment adherence, and improving patient health outcomes. Consistent with these models, the Patient Protection and Affordable Care Act (PPACA, 2010) identifies patient engagement as central to disease management, prevention, and shared decision-making between provider and patient regarding treatment options [22].

Despite the emphasis of care models based on patient engagement, to our knowledge no comprehensive data-driven, systematic reviews of patient engagement have been published. If poor patient engagement proves to be a risk factor for worse chronic disease outcomes, early identification of individuals who are low in patient engagement coupled with tailored behavioral interventions to enhance participation may provide a reasonable and low cost target for personalized health care innovation. In fact, the role of patient engagement in risk and outcome prediction may well be among the most important and modifiable risk factors for chronic disease outcomes and thus needs to be further elucidated. Moreover, the specific processes within patient-provider interactions that increase patients’ motivation, authority, and ability to participate in their care to promote long-lasting health need clear articulation [23-25]. The purpose of this article is to provide such a review with an eye toward implications for incorporating patient engagement as a risk factor in health risk appraisals to enhance personalization for chronic disease care. Such a review is essential as efforts continue to transform healthcare delivery from a reactive, disease-based system to one that is proactive, preventive, and personalized. Specifically, it is essential to understand the degree to which patients have the knowledge and skills to engage in their care and use that knowledge to perform health-promoting behaviors. Systematic review of these data and their link to disease outcomes is particularly needed for the most prevalent chronic diseases, where prevention and management still are largely centered on behavioral strategies ranging from diet, exercise, and stress reduction to medication adherence and home disease monitoring. Indeed, some evidence suggests that patient activation scores measuring knowledge, skills, and confidence to manage health significantly predict healthcare costs, even after controlling for Episode Risk Groups®, a risk score for cost prediction [26].

The purpose of this review was to systematically examine the relationship between patient engagement and health outcomes in chronic disease. The term 'patient engagement' is used to reflect a tripartite definition: (1) recognizing and understanding the importance of taking an active role in one’s health and health care; (2) having the knowledge, skills, and confidence to manage health; and (3) using knowledge, skills and confidence to engage in health-promoting behaviors to obtain the greatest benefit [27,28]. Specifically, to examine whether patient engagement improves chronic disease outcomes, we reviewed randomized, controlled trials that assessed the efficacy or effectiveness of interventions designed to engage patients in their care to improve patient engagement and health outcomes compared with an appropriate control in adults with chronic disease.

## Materials and methods

### Search strategy

This search covered 20 years of peer-reviewed literature, confined to English language articles published between January 1993 and December 2012, including e-publications ahead of print. A medical librarian searched Medline, Embase, and Cochrane databases using the following search terms: 'self care'[mesh] OR 'self care'[tiab] OR self manag*[tiab] OR self monitor*[tiab] OR 'decision making'[mesh] OR 'decision making'[tiab] OR shared decision*[tiab] OR 'patient education as topic'[mesh] OR 'patient education'[tiab] OR 'motivation'[mesh] OR 'motivation'[tiab] OR patient motivat*[tiab] OR 'patient participation'[mesh] OR 'patient participation'[tiab] OR patient participant*[tiab] OR patient engage*[tiab] OR patient activat*[tiab] OR patient involve*[tiab]. Searches were limited to randomized clinical trials, meta-analyses, and systematic reviews of intervention studies among adults aged 18 years and older with chronic disease. The last search was run 30 September 2013.

### Study selection

The following inclusion criteria were defined: (1) a prospective, randomized clinical trial with minimum 3 months duration between pre- and post-intervention measurement of outcomes; (2) the trial investigated an intervention designed to increase patient engagement; (3) patient engagement was measured individually or as a composite of multiple measures assessing knowledge, skills, confidence, and behaviors; and (4) clinical or self-reported health outcomes were measured. Exclusion criteria included: (1) trials targeting psychiatric disorders, given that mental functioning has a significant influence on the capacity for individuals to engage in health-promoting behaviors; (2) trials targeting providers and caregivers rather than patients themselves; and (3) trials of pediatric subjects. Three researchers (LAS, RQW, and EMB) and two research assistants screened titles and abstracts for relevant studies to preliminarily assess inclusion/exclusion criteria. Articles deemed relevant or possibly relevant were obtained and evaluated to confirm eligibility, including a review of study instruments used to assess the components of patient engagement. A data collection form was developed for members of the research team to independently record information on study methodology and results. Data included the following: authors, year published, country of origin, blinding method (if any), mean age and gender of participants at enrollment, total sample size, inclusion criteria, definition and measurement tool(s) for patient engagement, intervention type (for example, online, in person, group, individual) and length, control type and length, trial duration (time between baseline and final follow-up measure), outcome measures, 95% confidence intervals, and significance levels based on multivariate regression models where available. Two investigators (LAS and RQW) used the form to remove all papers not meeting the prescribed criteria. Disagreements were resolved by consensus with another investigator (RS).

### Data analysis

Due to significant heterogeneity among study designs, chronic diseases studied, and measures of patient engagement, a robust meta-analysis was impossible to conduct. Even for trials where the primary disease was the same, inconsistent measures of patient engagement and diverse intervention strategies prevented robust statistical analysis. Thus, a qualitative summary of the findings is presented. As part of this summary, we described included studies according to the following data fields: chronic disease studied, intervention type and length, control type, patient engagement measure(s), significant clinical outcomes grouped by chronic disease. However, to assess the validity and methodological quality of eligible articles, Jadad scores [29] were calculated for all studies. Scoring includes ratings of adequacy and concealment of randomization, blinding, power and sample size calculations, and whether analyses accounted for loss to follow-up.

## Results

**Figure 1:**
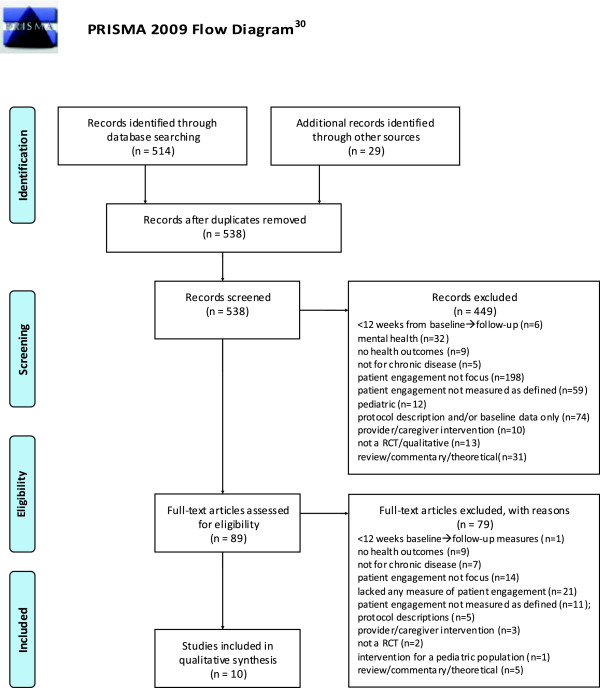
**PRISMA 2009 flow diagram **[30]. RCT, randomized controlled trial.

Figure 1 documents the flow for the systematic review. A total of 10 studies met inclusion criteria for the review using the following process. The keyword search identified a total of 514 studies. Additionally, 29 articles were identified from the references of meta-analyses and systematic reviews on hypertension, diabetes, and chronic care. After adjusting for duplicates, 538 articles remained. Title and abstract screening resulted in 449 articles being excluded with reasons, leaving 89 potentially relevant articles, which were retrieved for full-text review. Data were abstracted using the data-reporting sheet. Of these, 79 were excluded for multiple reasons, leaving 10 articles for the analysis. No unpublished relevant studies were obtained.

Table 1 summarizes the 10 studies included in the primary analysis [31-40]. Five studies focused on diabetes [32,37-40], and there was one study each on individuals with various chronic diseases (for example, hypertension, diabetes, coronary artery disease, and so on) [34], multiple sclerosis (MS) [31], arthritis [33], asthma [35], and bronchiectasis [36]. Six studies [34-39] (60%) compared the intervention to usual care or no active control, while the remaining four studies (40%) compared the intervention to an attention control [33] (n = 1), enhanced usual care [32] (n = 1), or a wait-list control [31,40] (n = 2). Half of the interventions were in-person group-based workshops [31,33,34,36,38] (n = 5); the remaining interventions were in person one-on-one sessions [35,39] (n = 2), internet-based modalities [32,37] (n = 2), and personal telephonic coaching [40] (n = 1). Intervention lengths ranged from weekly 2-hour sessions for 6 weeks to 24 months. Four of the 10 studies [33,35,36,39] (40.0%) were high quality based on Jadad scoring (score ≥3) (Table 2).

**Table 1:** Summary of included trials

**Study (year)**	**Disease or condition**	**N**	**Mean age (SD)**	**Sex**	**Intervention**	**Control**	**Intervention length**	**Follow up**	Results*
Barlow *et al.* (2009) [31]	MS	216	I = 48.2 (10.1)	59 M	In-person group-based chronic disease self-management program	Wait-list control + comparison group	6 weeks (2 h/week)	4, 12 months	0 Self-management self-efficacy (*P* = 0.132, ES = 0.30)
			C = 50.7 (11.7)						+ MSIS physical status (*P* = 0.005, ES = 0.21)
			Comparison = 54.6 (10.8)						0 Improvement in depression (*P* = 0.632)
									0 MS self-efficacy (*P* = 0.161)
									0 cognitive symptom management (*P* = 0.140)
									0 MD communication (*P* = 0.861)
Glasgow *et al.* (2012) [32]	Diabetes	463	I (CASM+) = 57.8 (9.3)	232 M	Internet-based diabetes self-management program with (CASM+) and without enhanced social support (CASM)	Enhanced usual care (computer-based health risk appraisal feedback with recommendations for preventive behaviors)	12 months (ongoing internet-based intervention); CASM + group also received three 120-minute group sessions and two follow-up calls	4, 12 months	+ Health behaviors (eating habits, fat intake, physical activity: *P* < 0.05, ES = 0.09-0.16)
			I (CASM) = 58.7 (9.3)						+ Biological outcomes (lower HbA1c, improved lipid ratio, BP MAP, 10-year CHD risk: *P* < 0.05)
			C = 58.7 (9.1)						+ Psychosocial and QOL measures (self-efficacy, problem solving, general health state, diabetes distress: *P* < 0.05)
									0 Medication adherence
Goeppinger *et al.* (2007) [33]	Arthritis	416	I = 64 (12.78)	75 M	Arthritis self-help group: small group, in-person workshops specific to arthritis	Generic chronic disease self-management group: small group, in-person workshops not specific to arthritis	6 weekly sessions 2–2.5 h each	4, 12 months	+ Self-efficacy (*P* = 0.004)
			C = 64 (12.8)						
									+ General health (*P* = 0.016)
									+ Stretching minutes (*P* = 0.023)
									+ Strengthening minutes (*P* = 0.016)
Hibbard *et al.* (2007) [34]	At least one of six chronic diseases (T2D, HTN, arthritis, CHD, COPD, hyperlipidemia)	479	I = 59.6	147 M	In-person group-based chronic disease self-management program	No intervention	6 weeks	6 months	0 Engagement (PAM) (*P* < 0.001)
			C = 60.0				(2.5 h/1 wk)		
									+ General self-management behaviors (*P* < 0.05)
									+Diabetes self-management behaviors (*P* < 0.05)
									+Arthritis self-management behaviors (*P* < 0.05)
									+ HRQoL (*P* = NR)
Huang *et al.* (2009) [35]	Asthma	148	I = NR	108 M	Individualized self-care education program, with and without peak flow monitoring (PFM)	Usual care	6 months	1, 6 months	+ Asthma self-care competence (*P* < 0.001)
			I + PFM = NR						+ Asthma self-care behaviors (*P* < 0.001)
			C = NR						Asthma self efficacy (*P* < 0.001)
									0 Unscheduled health service usage
Lavery *et al.* (2011) [36]	Bronchiectasis	64	I = 60 (9)	29 M	In-person, group-based patient self-management program	Usual care	8 weeks (2.5 h/week)	3, 6 months	+ Self-efficacy in exercise (*P* = 0.02); to get information about disease (*P* = 0.03); to manage disease in general (*P* = 0.05); to do chores (*P* = 0.04); for social/recreational activities (*P* = 0.03); to manage symptoms (*P* < 0.01); to control/manage depression (*P* = 0.01)
									0 Self-efficacy in obtaining help from community, family and friends (*P* = 0.06); communicate with physician (*P* = 0.85); to manage shortness of breath (*P* = 0.08)
									+ Symptom reporting (*P* < 0.05)
									+ Decreased QOL (*P* = 0.01)
									+ Increase in self-reported health care use (*P* < 0.05)
									0 IPQ-R score
									0 Lung function
			C = 60 (8)						
Lorig *et al.* (2010) [37]	Diabetes	761	All = 54.3	206 M	Internet-based diabetes self-management program	Usual care	6 weeks	6, 18 months	+ Engagement (PAM) (*P* = 0.021)
									+ Self-efficacy (*P* < 0/001)
									+ Lower HbA1C (*P* < 0.05; ES = 0.111)
									+ Lower HbA1C high subgroup baseline ≥7.0 (*P* = 0.01; ES = 0.499)
									0 Health behavior and utilization
									0 Exercise (*P* = 0.810)
Lorig *et al.* (2009) [38]	Diabetes	345	I = 67.7 (11.9)	124 M	Community-based, peer-led diabetes self-management program	Usual care	6 weeks	6, 12 months	+ Engagement (PAM) (*P* = 0.017)
							(2.5 h/1 wk)		
			C = 65.4 (11.4)						+ Self-efficacy (*P* = 0.001)
									0 Lower HbA1C
									+ Hypoglycemia symptoms (*P* = 0.002; ES > 0.30)
									0 Hyperglycemia symptoms
									+ Healthy eating (*P* < 0.001; ES > 0.30)
									+ Lower depression (*P* < 0.001)
									+ Communication with providers (*P* = 0.016)
Moriyama *et al.* (2009) [39]	Diabetes	75	I = 66.4 (9.2)	30 M	In person, individual self-management education program	Usual care	12 months	3, 6, 9, 12 months	+ Lower body weight (*P* = 0.001)
									+ Lower HbA1C (*P* = 0.049)
									+ Self-efficacy (*P* = 0.001)
									+ Dietary and exercise stages (*P* = 0.017 and *P* = 0.020)
									+ Degree of goal attainment (*P* = NR)
									+ QOL (*P* = 0.055)
									+ Lower diastolic BP (*P* = 0.067)
									+ Lower total cholesterol (*P* = 0.087)
			C = 65.2 (8.5)						
Wolever *et al.* (2010) [40]	Diabetes	56	I = 53.1 (8.29)	13 M	Integrative health coaching	Wait-list control	6 months	1 month	+ Engagement (PAM) (*P* < 0.001)
			C = 52.8 (7.64)				(14 sessions, 0.5 h each)		+ Medication adherence (*P* < 0.005)
									+ Perception of illness (*P* < 0.05)
									+ Psychosocial health (*P* < 0.05)
									0 Lower HbA1C (all subjects)
									+ Lower HbA1C for high subgroup baseline >7.0 (*P* = 0.016; ES = 0.34)

BP, blood pressure; BP MAP, blood pressure mean arterial pressure; C, control; CASM, computer-assisted self-monitoring; CHD, chronic heart disease; COPD, chronic obstructive pulmonary disease; ES, effect size; HRQOL, health-related quality of life; HTN, hypertension; I, intervention; IPQ-R, Illness Perception Questionnaire-Revised; M, male; MS, multiple sclerosis; MSIS, Multiple Sclerosis Impact Scale; NR, not reported; PAM, Patient Activation Measure; PFM, peak flow monitoring; QOL, quality of life; SD, standard deviation; T2D, type 2 diabetes.

**Table 2:** Summary of methodological quality rating

**Study**	**Country**	**Design**	**Rationale described?**	**Power calculation presented?**	**Sample size calculations presented?**	**Selection criteria described?**	**Adequate concealment of randomization?**	**Assessor blinded?**	**Intention to treat analysis performed?**	**Quality score**	**Quality rating**
Barlow *et al.*[31]	UK	Parallel	Yes	No	No	Yes	Yes	NR	Yes	2	Low
Glasgow *et al.*[32]	USA	3-arm	Yes	Yes	Yes	Yes	Yes	NR	Yes	2	Low
Goeppinger *et al.*[33]	USA	Parallel	Yes	Yes	Yes	Yes	Yes	NR	NA	3	High
Hibbard *et al.*[34]	USA	Parallel	No	No	No	Yes	NR	NR	No	1	Low
Huang *et al.*[35]	Taiwan	3-arm	Yes	Yes	Yes	Yes	Yes	Yes	No	4	High
Lavery *et al.*[36]	Ireland	Parallel	Yes	No	No	Yes	Yes	Yes	Yes	3	High
Lorig *et al.* (2010) [37]	USA	Parallel	No	No	No	Yes	NR	NR	Yes	2	Low
Lorig *et al.* (2009) [38]	USA	Parallel	Yes	No	No	Yes	NR	NR	Yes	2	Low
Moriyama *et al.*[39]	Japan	Parallel	Yes	No	No	Yes	NR	NR	No	3	High
Wolever *et al.*[40]	USA	Parallel	Yes	No	No	Yes	NR	Yes	No	2	Low

NA, not applicable; NR, not reported.

### Patient engagement

Four [34,37,38,40] of the 10 studies (40.0%) measured the knowledge, confidence, and skills components of patient engagement using the Patient Activation Measure (PAM) [41], a well-validated instrument. Five studies [31-33,35,36] (50%) used disease-specific or chronic disease measures of self-efficacy, and one study [39] (10%) used a general measure of self-efficacy. Most studies had multiple behavioral measures (for example, medication adherence, physical activity, nutrition/eating, goal attainment, symptom management), while only Barlow *et al.*’s [31] study of MS had a single measure of the behavioral component of engagement. Nine [32-36,38-40] of the 10 studies (90.0%) reported improvements in all components of patient engagement (knowledge, skills, confidence, and at least one behavior). Only one study [31] reported no changes in any component of patient engagement, and one study reported improvements in knowledge/confidence/skills but not behavior [37].

### Clinical outcomes: diabetes

Each of the five studies that investigated interventions for diabetes reported improvements in all components of patient engagement. Additionally, each study examined one primary biological outcome (HbA1C) as well as other biological (for example, lipids, blood pressure) and health-related (for example, self-rated health, diabetes distress) outcomes. Three [32,37,39] of the five studies reported a significant reduction in HbA1C for all intervention subjects compared with controls. A second [40] found a significant reduction in HbA1C for intervention subjects who enrolled with elevated baseline levels (>7.0). Only one study [38] reported no differences between intervention participants and controls on HbA1C, although significant improvements were noted on other outcomes of interest, including symptoms of hypoglycemia, depression, or indices of healthy eating and communication with providers. In addition to HbA1C, two studies [32,39] found improvements in lipid ratios, blood pressure, and total cholesterol among intervention participants compared to controls. Four [32,38-40] of the five studies also noted improvements in at least one lifestyle behavior (for example, physical activity, healthy eating/nutrition, medication adherence), while two studies [32,39] reported improvements in health-related quality of life, and one study each reported reduced diabetes distress [32] and improved perception of illness [40].

### Clinical outcomes: other chronic diseases

Of the remaining five studies that reported on interventions for various chronic diseases, only two [35,36] investigated the effect of the intervention on clinical outcomes. Huang *et al.*[35] found that the intervention improved lung function for asthma, noting significant differences in prebronchodilation FEV1 (forced expiratory volume in one second) and FVC (forced vital capacity). Lavery *et al.*[36] assessed lung function in patients with bronchiectasis, but reported no differences in clinical outcomes between the intervention and control groups despite noted improvements in patient engagement. The remaining three studies examined health outcomes using self-reported assessments. Barlow *et al.*’s [31] intervention for MS reported improvements in health-related quality of life as assessed by the Multiple Sclerosis Impact Scale. In their intervention for patients with arthritis, Goeppinger *et al.*[33] demonstrated improvements in self-reported general health status. Hibbard *et al.*’s [34] study of patients with various chronic diseases reported improvements in health-related quality of life. Huang *et al*.’s [35] study of asthma found no differences in unscheduled health services utilization despite improvements in lung function, although they did note improved asthma control for participants in the group that actively monitored peak flow readings. In Lavery *et al*.’s [36] study of bronchiectasis they found improvements in quality of life and symptom reporting, despite no changes in lung function.

### Secondary analyses

Given the limited number of studies that met the inclusion criteria for this review, we conducted a qualitative secondary analysis of 11 additional trials [14,42-51] that met all of the inclusion criteria with one exception: the measure of patient engagement lacked one of the three key *a priori* components (data not shown). Specifically, seven studies [14,43,46-50] (63.6%) did not measure knowledge, one study did not measure confidence [42] (9.1%), and three studies [44,45,51] (27.3%) did not measure any health behaviors; six [43,44,47,48,50,51] of these studies (54.5%) were of high quality. We described these studies according to the same data fields as described in the primary analyses above. Of note, seven [14,43,45,47-50] of the studies (63.6%) were not disease-specific, but included individuals with at least one of various chronic diseases; one trial was for low back pain [44], one for hypertension [51], one for heart failure [42], and one for arthritis [46]. The majority of these interventions was also brief, with eight [14,43-47,49,50] (80%) lasting 7 weeks or less and the remaining three (25%) lasting 6, 12, and 26 months, respectively [42,48,51]. Among the seven trials that did not include a measure of knowledge [14,43,46-50], there were significant improvements observed for confidence in skills and health-promoting behaviors among intervention subjects compared with controls. Each of these studies also documented improvements in one or more measures of self-rated health. The trial that did not measure confidence [42] showed no significant differences in health behaviors or health-related quality of life, despite increases in knowledge. Among the three trials that did not measure specific behaviors [44,45,51], there were no documented improvements in confidence in skills or knowledge and only one study demonstrated improvements in self-rated health (emotional well-being).

## Discussion

The role of patient engagement in chronic disease care is increasingly being cited as critical for improving health outcomes and reducing costs. In this review, patient engagement was defined as: (1) understanding the importance of taking an active role in one’s health; (2) having knowledge, skills, and confidence to manage health/chronic conditions; and (3) performing health-promoting behaviors. Intervention studies that aimed to increase patient engagement were examined to determine if they indeed did so, and if they impacted health outcomes. Four main findings were identified. First, it was quite surprising how few studies that aim to engage patients actually quantify and measure patient engagement. Second, this review supports the link between patient engagement and improved outcomes. Third, relatively short interventions can increase patient engagement and promote positive health outcomes. Fourth, specific behavioral targets and their tracking appear essential to assess the role of patient engagement over time to achieve positive health outcomes.

Given the enormous emphasis of policy and legislation on patient engagement, it was surprising to find only 89 relevant randomized controlled trials, and to further find that 21 of those had no quantifiable measure of patient engagement at all. This dearth is particularly concerning given the burgeoning policy implications of the Affordable Care Act and design of medical home models that leverage patient engagement [15,16]. We call forth researchers to immediately address this major gap in the literature.

On the positive side, although there are relatively few randomized controlled trials of interventions that aim to increase patient engagement as defined *a priori*, with one exception [31], those interventions did demonstrate a positive link between engagement and health outcomes. Moreover, nearly half of these studies [33,35,36,39] were of high quality per Jadad scoring, despite being behavioral interventions where blinding is more challenging. Additionally noteworthy, four of the five studies that measured clinical outcomes reported improvements, including four studies that documented reductions in HbA1C [32,37,39,40]. Two of these same studies also reported improvements in blood pressure [32,39], and two reported lower cholesterol levels [32,39]. Even those studies that did not assess disease biomarkers found improvements in self-rated health status.

Additionally important, findings show relatively short interventions (6 to 8 weeks for 2 to 3 hours per week) can be effective at increasing engagement and promoting positive health outcomes. As technology continues to evolve, there may be increased capabilities to supplement and/or supplant these interventions with mobile applications, interactive personalized health records, virtual environments, and the like [52-54]. These technologies can serve as a platform for increasing the capacity for patient engagement, especially those that facilitate the patient’s input of real-time clinical data and provider’s response with real-time, personalized recommendations based on those data. Moreover, as we become better able to document and track clinical and behavioral data and link them to intervention strategies through electronic health records, behavioral phenotyping [55-57] may be used to stratify individuals based on their risk for not responding to a particular intervention, and we can provide more intensive, personalized care to these individuals.

Finally, secondary analyses support our selection of inclusion criteria in the definition of patient engagement. Specifically, secondary analyses emphasize the importance of measuring behavior as a component of patient engagement. Those studies that did not measure behaviors had limited positive results despite their focus on and measurement of participants’ knowledge, skills, and confidence to manage health. On the other hand, studies that measured behaviors along with having confidence and skills (but not knowledge) demonstrated significant improvements not only in those components of patient engagement, but also in health outcomes. Not surprisingly, the study that measured knowledge, but not confidence or skills, did not see improvements in health outcomes either. These findings underscore the role of behavioral tracking as a central feature of patient engagement, and strategies to enhance patient participation in their health should include this key element [24,58-60]. Additionally, they bring to the forefront the frequently observed gap between 'knowing' what to do and actually doing it, and suggest that strategies for building confidence in applying knowledge are essential to bridging the 'know-do gap' for successful behavioral management of health.

That relatively short interventions link to positive increases in patient engagement and chronic disease outcomes is good news when considering incorporation of patient engagement as a risk factor in health risk appraisals. Indeed, evidence from this review suggests that patient engagement should be considered a risk factor for current and future health status as a component of a comprehensive, personalized approach to care. Moreover, it is essential that currently available and emerging data be used to support the design and implementation of interventions that (1) increase patients’ knowledge, skills, and confidence to engage in health-promoting behaviors; and (2) result in improved health.

There are challenges to including patient engagement as part of a health risk appraisal, as this review demonstrates. First, there is considerable variability in quantifying engagement, and in this review the construct was measured using multiple instruments. One standardized and well-validated measure of engagement observed in the literature was the PAM [41]. Specifically, the PAM identifies engagement as consisting of four broad constructs with associated beliefs and behaviors: (1) believing one’s role as an active patient is important; (2) having confidence and knowledge to take action; (3) taking action to maintain and improve one’s health; and (4) staying the course even under stress [41]. Many of the studies in this review [31-33,35] used disease-specific scales of self-efficacy, health knowledge, and health behaviors. Given the various definitions that exist for patient engagement, further theoretical and definitional work may be needed to fully develop what constitutes an 'engaged patient' and to measure it reliably. However, given that the PAM [41] is a valid and reliable instrument, it may be that the PAM plus some number of agreed upon minimum behaviors (for example, physical activity, eating behaviors/nutrition, sleep efficiency, stress management practices, medication adherence, and home monitoring of a given biomarker) is a reasonable start for the majority of the population that can be implemented immediately with current tools and serve to effectively personalize care for chronic disease prevention and management.

## Conclusions

While this review demonstrated the positive effects of disease management interventions on increasing patient engagement and improving health outcomes, more studies are needed. Less than half of the studies in this review included a biological marker of disease. Future research should include large-scale studies based on stringent methodology that utilize well-validated measures of all components of engagement in addition to biological markers of disease progression. These will offer more robust evidence for not only the effects of patient engagement, but also the characteristics of interventions that increase engagement and associated behaviors. Unfortunately, the challenges to implementing such trials are significant. First, trials need to be of extensive duration (at least one year) to adequately document the process and time course in a way that captures behavioral change-driven biological shifts that predict morbidity and mortality. Second, such trials also need to be large enough to study potential confounding factors (for example, interactions with conventional medical treatments, including medication) and identify those factors most critical to producing desired health outcomes. Hence, comparative effectiveness trials are likely next candidates to bring methodological rigor to the study of engagement interventions. Such studies will further benefit from inclusion of cost-effectiveness measures both in terms of healthcare utilization and expenditures as well as measures of individual productivity (at work or in society). No such studies were evident in our review of the literature.

Whether in studies or purely clinical venues, the design of comprehensive, personalized approaches to care that engage patients to prevent disease and manage their health also will benefit from a number of considerations gleaned from our systematic review that prevented us from conducting a robust meta-analysis, and thus, are limitations. First, the diversity of interventions across varying chronic diseases made it difficult to summarize findings using statistical analyses. This points to the need for interventions to consistently provide a clear delineation of length and frequency of contact, type of contact (for example, web-based, telephonic, in person, individual, group), training and expertise of providers, tracking and planning tools, and durability of engagement over time. Indeed, though our findings suggest that short interventions can improve patient engagement and health outcomes, these improvements may be related to the Hawthorne effect and the fact that study participants improved simply because they were being observed [61]. Similarly, the potential contribution of interpersonal support alone to enhance behavioral change can not be dismissed in these trials. Moreover, there is some evidence to suggest that patients who enroll in studies are more engaged at the outset and will thus do better regardless compared to a less engaged individual who does not enroll [62]. However, the studies in our review noted improvements across ranges of starting activation levels, suggesting that irrespective of baseline engagement levels, with the appropriate (personalized) support, increased engagement is both possible and beneficial. Finally, given the diversity of measurements for patient engagement, we were unable to conduct any statistical analyses or assessments of bias; however, our secondary analyses, which included a less stringent definition of patient engagement, suggest our findings to be consistent with the available literature. Clearly, more data are necessary to determine whether engagement interventions need to be disease-specific, as was the case for many of the studies in this review, and whether engagement approaches work equally well across different behaviors (for example, medication adherence, provider communication, diet, physical activity, stress management) if adequate personalization occurs. While these endeavors are significant undertakings, given the growing burden of chronic disease in the United States, they are critical to the future health of the population. Meantime, providers can begin to incorporate assessment of patient engagement into current care practices to enhance personalization and align their recommendations with patients’ needs for information, confidence, and enacting skills. These assessments also can help to provide needed data on patient engagement in non-research clinical settings.

As personalized health care is implemented for chronic disease prevention and management, patient engagement that considers knowledge, confidence, skills, and behaviors is a critical risk factor to measure and track over time. Having an understanding of how engaged patients are, where patients’ specific challenges exist (for example, they may 'know' what to do but they do not have the confidence to do what they know, or there may be a mismatch between what the patient understands about their health and their actual current and predicted health status), and how these factors change over time can aid clinicians in personalizing care within the context of patients’ real lives. That is, it can help both clinicians and patients to understand what support, information, skills training, and other resources are needed for successful health management. This patient-provider alignment will not only enhance the provision of personalized health care, but with consistent implementation also will contribute to improved health outcomes and reduced healthcare expenditures over time.

## Abbreviations

MS: multiple sclerosis; PAM: Patient Activation Measure.

## Competing interests

LAS has funding from NHLBI/NIH, the Duke Center for Personalized and Precision Medicine, and the Veteran’s Health Administration through the Pacific Institute for Research and Evaluation. She also provides health coaching to private clients and consulting to healthcare providers on patient engagement strategies in clinical care. RW has funding from NHLBI/NIH, NIDCD/NIH, the Duke Center for Personalized and Precision Medicine, the United States Air Force, the Bravewell Philanthropic Collaborative and Nurtur. She serves as the Chief Scientific Advisor to eMindful and provides consultation to industry related to health behavior change. EMB declares that she has no competing interests. RS is on the Boards of Crescendo Biosciences and XDC, Inc.

## Authors’ contributions

LAS conceived and coordinated the study, and participated in design, data abstraction, data analysis, results interpretation, and drafting the manuscript. RQW participated in study design, data abstraction, data analysis, results interpretation, and drafting the manuscript. EMB participated in data abstraction, data analysis, and table compilation. RS participated in study design, data analysis, results interpretation, and drafting the manuscript. All authors read and approved the final manuscript.
